# Biological Activity of Hexaazaisowurtzitane Derivatives

**DOI:** 10.3390/molecules28248084

**Published:** 2023-12-14

**Authors:** Daria A. Kulagina, Sergey V. Sysolyatin, Svetlana G. Krylova, Elena A. Kiseleva, Tatiana N. Povetyeva, Elena P. Zueva, Valeria V. Eremina, Natalia A. Alekseeva, Svetlana V. Strokova, Nikolai I. Suslov, Vadim V. Zhdanov

**Affiliations:** 1 Laboratory for Medicinal Chemistry, Institute for Problems of Chemical and Energetic Technologies, Siberian Branch of the Russian Academy of Sciences (IPCET SB RAS), 659322 Biysk, Russia; dir@ipcet.ru (S.V.S.); eremina_v.v@mail.ru (V.V.E.); bna19.84@mail.ru (N.A.A.); sveta.ru.1997@bk.ru (S.V.S.); 2 Goldberg Research Institute of Pharmacology and Regenerative Medicine (GRIP&RM), Tomsk National Research Medical Center, Russian Academy of Sciences, 634028 Tomsk, Russia; krylova5935@gmail.com (S.G.K.); pupsyalenchik@gmail.com (E.A.K.); ptn@bk.ru (T.N.P.); zep0929@mail.ru (E.P.Z.); nis-51@mail.ru (N.I.S.); zhdanov_vv@pharmso.ru (V.V.Z.)

**Keywords:** hexaazaisowurtzitane, analgesic activity, anti-inflammatory activity, anticonvulsant activity, toxicity

## Abstract

Biologically active compounds of natural or synthetic origin have a complex structure and generally contain various structural groups among which polycyclic cage amines are found. Hexaazaisowurtzitanes are representatives of these amines and studies on their biological activity began less than two decades ago, starting with research on the environmental impact of CL-20. This research helped to evaluate the risks of potential pollution in the habitat environments of living organisms and determine whether the chemical compounds in question could be utilized in pesticides, herbicides, fungicides, or medicinal drugs. The nomenclature of hexaazaisowurtzitane compounds has recently been expanded significantly, and some of them have demonstrated promise in the design of medicinal drugs. This paper review studies the pharmacological activity of the acyl derivatives of hexaazaisowurtzitane. Most of the compounds have been found to possess a high analgesic activity, providing a solution to the pressing issue of pain management in current pharmacology. Analgesic drugs currently used in the clinical practice do not meet all of the efficacy and safety requirements (gastro-, nephro-, hepato-, haematotoxicity, etc.). The material presented in the seven sections of this paper highlights information about hexaazaisowurtzitane derivatives. Furthermore, they have been observed to exhibit anti-inflammatory, anticonvulsant, antihypoxic, and antimetastatic activities, which render them highly promising for evaluation in various fields of medicinal practice.

## 1. Introduction

**Figure 1:**
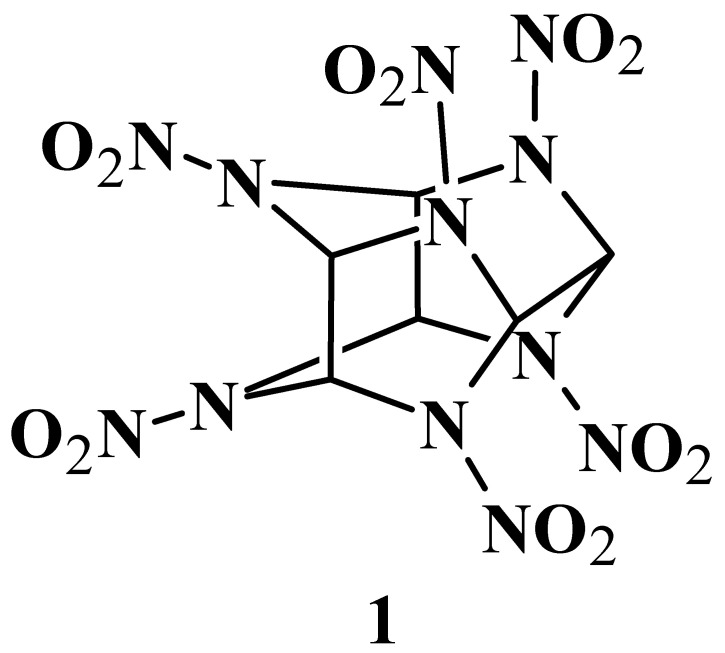
CL-20, an explosive from the class of hexaazaisowurtzitanes.

Hexaazaisowurtzitanes have emerged as a promising class of compounds for the industrial production of new substances [1,2,3]. Among them, CL-20 (**1**) is the most well-known representative and extensive studies have confirmed its xenobiotic nature (Figure 1). Toxicology studies investigating the effects of high doses of CL-20 on animal organisms have revealed that its negative impact, mediated through various biotransformation pathways, is mostly reversible [4,5,6,7,8,9,10,11,12,13,14,15,16,17,18]. Interestingly, current genetic research methods demonstrate the reversibility of the neurotoxic effects of compound **1** on *Eisenia fetida* [11]. In that study, a targeted RNA microarray chip designed for 15,119 unique transcripts of *Eisenia fetida* was developed. The obtained data enabled gene expression profiling in response to the influence of compound **1**. For this purpose, 18 earthworms were subjected to a 6-day exposure of 0.2 μg/cm^2^ of the compound, half of which were subsequently placed in a clean environment for a week. Nine control animals were euthanized on the sixth and thirteenth days of the experiment. Electrophysiological measurements revealed a significant reduction in the conduction velocity of the median giant nerve fiber after the 6-day exposure to compound **1**, but it was restored within 7 days after the exposure ceased. The statistical and bioinformatic analyses showed that compound **1** initiated neurotoxicity through the non-competitive blockade of the ligand-gated ion channel of the GABAA receptor, resulting in altered gene expression involved in the GABAergic, cholinergic, and agrin–muscarinic pathways. During the recovery phase, the expression of affected genes returned to normal, possibly through autophagy and the dissociation/metabolism of CL-20. In contrast, the use of the common cyclic nitramines, such as hexogen (RDX) and octogen (HMX), resulted in more severe and irreversible damage to the central nervous system (CNS) and the hematological systems of living organisms. The research findings on the nitro derivative of hexaazaisowurtzitane provide compelling evidence of its high bioactivity and its ability to undergo biotransformation within animal organisms.

**Figure 2:**
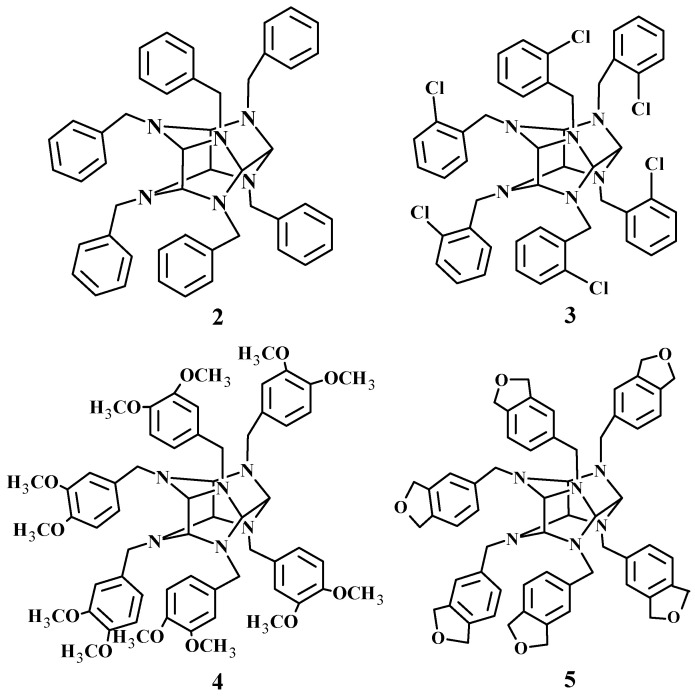
Cholinesterase inhibitors.

Initial studies on the biological activity of a series of hexaazaisowurtzitane compounds were conducted over 15 years ago. For instance, it was shown that 2,4,6,8,10,12-hexabenzyl-2,4,6,8,10,12-hexaazaisowurtzitane (2,4,6,8,10,12-hexabenzyl-2,4,6,8,10,12-hexaazatetracyclo[5.5.0.0^5,9^.0^3,11^]dodecane, **2**), 2,4,6,8,10,12-hexa(3,4-dimethoxybenzyl)-2,4,6,8,10,12-hexaazaisowurtzitane (2,4,6,8,10,12-hexa(3,4-dimethoxybenzyl)-2,4,6,8,10,12-hexaazatetracyclo [5.5.0.0^5,9^.0^3,11^]dodecane, **3**), 2,4,6,8,10,12-hexa(2-chlorobenzyl)-2,4,6,8,10,12-hexaazaisowurtzitane (2,4,6,8,10,12-hexa(2-chlorobenzyl)-2,4,6,8,10,12-hexaazatetracyclo [5.5.0.0^5,9^.0^3,11^]dodecane, **4**), [19] and 2,4,6,8,10,12-hexa(3,4-methylenedioxyphenylmethyl)-2,4,6,8,10,12-hexaazaisowurtzitane (2,4,6,8,10,12-hexa(3,4-methylenedioxyphenylmethyl)-2,4,6,8,10,12-hexaazatetracyclo [5.5.0.0^5,9^.0^3,11^]dodecane, **5**) represent potent, peripherally restricted anionic inhibitors of acetylcholinesterase and butyrylcholinesterase [20], with compounds **2–4** being more selective towards butyrylcholinesterase rather than acetylcholinesterase (Figure 2).

**Figure 3:**
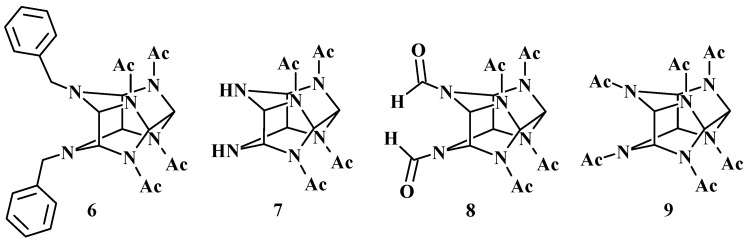
Isowurtzitane compounds displaying anticonvulsant activity.

In 2010, it was demonstrated that 4,10-dibenzyl-2,6,8,12-tetraacetyl-2,4,6,8,10,12-hexaazaisowurtzitane (4,10-dibenzyl-2,6,8,12-tetraacetyl-2,4,6,8,10,12-hexaazatetracyclo [5.5.0.0^5,9^.0^3,11^]dodecane, **6**), 2,6,8,12-tetraacetyl-2,4,6,8,10,12-hexaazaisowurtzitane (2,6,8,12-tetraacetyl-2,4,6,8,10,12-hexaazatetracyclo [5.5.0.0^5,9^.0^3,11^]dodecane, **7**), 4,10-diformyl-2,6,8,12-tetraacetyl-2,4,6,8,10,12-hexaazaisowurtzitane (4,10-diformyl-2,6,8,12-tetraacetyl-2,4,6,8,10,12-hexaazatetracyclo [5.5.0.0^5,9^.0^3,11^]dodecane, **8**), and 2,4,6,8,10,12-hexaacetyl-2,4,6,8,10,12-hexaazaisowurtzitane (2,4,6,8,10,12-hexaacetyl-2,4,6,8,10,12-hexaazatetracyclo [5.5.0.0^5,9^.0^3,11^]dodecane, **9**) exhibit anticonvulsant activity (Figure 3). Among these compounds, compound **7** demonstrated the most significant activity by completely preventing the corazole-induced blockage of GABA receptors and acting as a GABA mimetic [21]. These studies have laid the foundation for further drug design and directed synthesis of novel isowurtzitane compounds with biological activity, as well as for the development of efficacious and low-toxicity medicinal drugs derived from them. 

The structure of compound **7** provides an opportunity to explore a wider range of compounds by incorporating substituents on the amino groups. Previously, the predominant method for synthesizing 4,10-substituted 2,6,8,12-tetraacetyl-2,4,6,8,10,12-hexaazaisowurtzitanes involved the transformation of compound **6**, while the acylation of the amino groups in compound **7** was understudied and was rather collateral to the studies which focused on the synthetic methods of compound **1** [22,23,24,25,26,27,28]. 

Numerous acyl derivatives obtained through the acylation of compound **6** have been synthesized to date [29]. However, the process of selecting compounds for biological activity evaluation based on “chemical structure modification” or “pharmacophore incorporation” principles has several limitations. A more rational approach involves the use of in silico methods, such as the PASS program package, to select compounds for evaluation [30,31]. 

**Figure 4:**
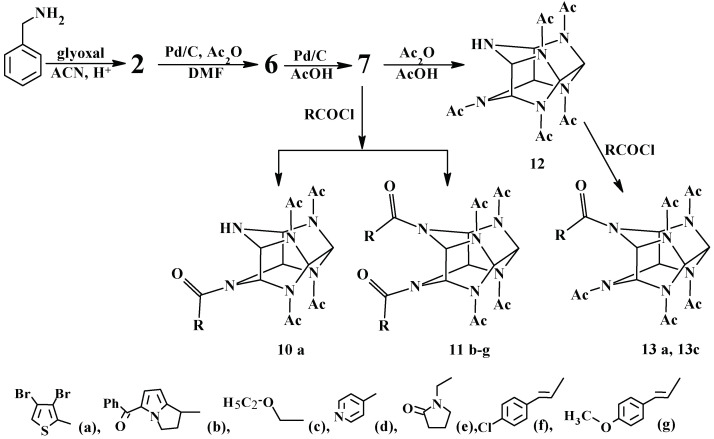
A general protocol for bioactive hexaazaisowurtzitane derivatives.

The basic synthetic method for bioactive acyl derivatives of hexaazaisowurtzitane relies on the acylation of compound **7** and is illustrated in brief in Figure 4. 

The screening of promising molecules using the PASS program package served as the basis for conducting a series of experiments to identify the in vivo pharmacological activity of the newly synthesized acyl derivatives of hexaazaisowurtzitane. 

The synthesized compounds underwent screening studies on animals, revealing a significant level of diverse bioactivities, including analgesic, anticonvulsant, anti-inflammatory, and antihypoxic properties.

## 2. Analgesic Activity of Hexaazaisowurtzitane Derivatives

Pain is one of the primary nocifensors that every person encounters repeatedly at any age. To combat pain syndromes of various etiologies, analgesic drugs from diverse pharmaceutical groups are used, ranging from non-steroidal anti-inflammatory drugs to opioid analgesics. Due to the demand for drugs in this pharmaceutical category, the development of substances with analgesic properties is considered a priority in medicinal chemistry [32,33]. The following widely used substances with analgesic activity were employed as reference drugs in the conducted studies: diclofenac sodium (active ingredient in medications such as Voltaren, Cataflam, etc.), which is used for inflammatory joint disease, neuralgias, myosalgias, post-traumatic pain syndromes, postoperative pains, migrainous strokes, and renal and biliary colic [34,35]; ketorolac tromethamine (active ingredient in medications such as Toradol, etc.), which is used for moderate and severe pain syndromes, including in the postoperative period, post-traumatic pain syndrome, neuralgias, and cancer pain syndrome [36,37]; tramadol (active ingredient in medications such as Tramadol, Ultram, etc.), which is used for moderate to severe pain syndromes (malignant tumors, traumas, and in the postoperative period) and as an anesthetic for diagnostic and therapeutic maneuvers [38,39]; and meloxicam (active ingredient in medications such as Mobic, etc.), which is used for inflammatory and degenerative joint diseases accompanied with pain syndrome, and neuralgias [40,41].

The first pharmacologically active compound among hexaazaisowurtzitanes patented is 4-(3,4-Dibromothiophenecarbonyl)-2,6,8,12-tetraacetyl-2,4,6,8,10,12-hexaazaisowurtzitane (4-(3,4-dibromothiophenecarbonyl)-2,6,8,12-tetraacetyl-2,4,6,8,10,12-hexaazatetracyclo [5.5.0.0^5,9^.0^3,11^]dodecane, **10a**) [42]. Its analgesic activity was initially discovered through the abdominal constriction test, which simulates acute visceral and deep somatic pain similar to clinical peritonitis in laboratory animals. In that study, outbred CD1 male mice were administered the test compound at doses of 50 and 100 mg/kg orally while diclofenac was given at a dose of 10 mg/kg daily over a 4-day period. On the final day, the administration was carried out 1 h before intraperitoneal injection of a 0.75% acetic acid solution. Diclofenac sodium, a non-steroidal anti-inflammatory drug, was selected as the reference drug, and its preventive administration resulted in a 33.6% reduction in pain response occurrences, as evidenced by a significant decrease in the number of writhes by a factor of 1.5 compared to the control group.

At a dose of 50 mg/kg, the test compound **10a** demonstrated a significant pain-relieving effect, reducing the occurrences of pain response by 56.9%. This was evident as the number of acetic acid-induced writhing decreased by 2.3 and 1.5 times, respectively, compared to the control group treated with a solvent and the group treated with diclofenac sodium. In the treatment group receiving a dose of 100 mg/kg, the pain response inhibition was 47.1%, which is comparable to the effect of diclofenac. The substantial reduction in pain severity, exceeding 50%, indicates the promising potential of compound **10a** for the development of analgesics based on its structure.

The study on a specific pharmacological activity on different experimental models of pain syndromes (under hyperalgesia, thermal, tonic and visceral pain, and adjuvant-induced arthritis) revealed a pronounced analgesic activity in compound **10a** when administered per os, comparable and/or exceeding that of analgesics from different groups, such as diclofenac sodium, ketorolac, tramadol, and meloxicam [43]. The analgesic action of **10a** was found to realize through different levels of nociceptive activity conduction and modulation, involving the system of central, suprasegmental, and peripheral neurophysiological mechanisms. After a prolonged course administration (28 days) of **10a**, no physical dependence or withdrawal symptoms were observed to develop. There were no signs of hyperalgesia, respiratory and cardiovascular disorders, or ulcerogenic effects typical of most analgesic groups. The evaluation of the neuropsychotropic properties of **10a** when administered for a prolonged time (28 days) demonstrated no sedation or psychostimulant action. These findings allow a highly confident prediction of the absence of the development of drug abuse during clinical trials of this potential analgesic. The neurochemical profile evaluation discovered that the test compound **10a** enhanced the GABA-ergic neurotransmission, diminished the serotonergic system activation, and is antimuscarinic. The test analgesic was found to exhibit a moderate anti-inflammatory action, with the ability to impact the production of arachidonic acid and bradykinin. All the evaluations on the action mechanism of **10a** were performed in vivo and in silico explain the absent morphine-like action and NSAIDs’ side effects by the fact that this analgesic mostly targets the TRPA1 and TRPV1 receptors, the voltage-dependent calcium channels [43].

Compound **10a** was also evaluated for pharmacokinetics, relative bioavailability, biotransformation, distribution across the organs and tissues, and excreted capsulated dosage form [44]. The agent was observed to be rapidly absorbed from the digestive tract, actively distributed throughout tissues and organs in rats, and exhibited a large distribution volume within the organism. Additionally, it demonstrated a high affinity for liver and kidney tissues. The study into the pharmacokinetic parameters in rats demonstrated the elimination constant to range from 0.54 to 0.68 1/h. The concentration achieved its maximum within 2 h in all cases of single administration. The mean retention time of the substance in the organism ranged from 5.67 to 17.15 post-injection. The pharmacokinetic study results after multiple injection of the agent at a therapeutic dose were shown to be similar to those after single intragastrical administration at the same dose, suggesting no accumulative effect. The agent is eliminated as the intact substance chiefly through the digestive tract at no more than 3.5% of the injected dose. Given the small absorption degree and low percentage of the excreted unaltered substance, the agent is presumed to have an active metabolism. 

The second registered substance is 4,10-bis((±)-5-benzoyl-2,3-dihydro-1H-pyrrolo [1,2-a]pyrrol-1-carbonyl)-2,6,8,12-tetraacetyl-2,4,6,8,10,12-hexaazaisowurtzitane (4,10-bis((±)-5-benzoyl-2,3-dihydro-1H-pyrrolo [1,2-a]pyrrol-1-carbonyl)-2,6,8,12-tetraacetyl-2,4,6,8,10,12-hexaazatetracyclo [5.5.0.0^5,9^.0^3,11^]dodecane, **11b**) [45]. An evaluation of its acute toxicity on outbred mice of both sexes revealed that this compound belongs to the category of low-toxicity substances, as the LD_50_ could not be determined. The analgesic activity of **11b** was assessed using the screening abdominal constriction test, with diclofenac sodium selected as the reference drug. At a dose of 50 mg/kg, compound **11b** reduced the severity of writhing pain reactions in outbred CD1 male mice by 39.1%. It significantly decreased the latency time for the development of the pain response compared to the control group. At a dose of 100 mg/kg, compound **11b** alleviated the pain response by 57.7%. This was achieved by suppressing the development of acetic acid-induced writhing by 2.4 times and 1.6 times, compared to the solvent-treated group and the group treated with diclofenac sodium. 

Further experiments have shown that the analgesic action of compound **11b** operates at different levels of nociceptive activity conduction and modulation, including suprasegmental (hot-plate test, Randall–Selitto test) and peripheral neurophysiological mechanisms (abdominal constriction test) [46]. 

The detected high analgesic activity and multitarget action of the investigated hexaazaisowurtzitane derivatives have enabled the expansion of pain genesis models to prove the analgesic effects of newly synthesized compounds, such as 4,10-di(ethoxyacetyl)-2,6,8,12-tetraacetyl-2,4,6,8,10,1-hexaazaisowurtzitane (4,10-di(ethoxyacetyl)-2,6,8,12-tetraacetyl-2,4,6,8,10,1-hexaazatetracyclo [5.5.0.0^5,9^.0^3,11^]dodecane, **11c**) [47,48], 4-(3,4-dibromothiophenecarbonyl)-2,6,8,10,12-pentaacetyl-2,4,6,8,10,12-hexaazaisowurtzite (4-(3,4-dibromothiophenecarbonyl)-2,6,8,10,12-pentaacetyl-2,4,6,8,10,12-hexaazatetracyclo [5.5.0.0^5,9^.0^3,11^]dodecane, **13a**) [49,50] and 4-(ethoxyacetyl)-2,6,8,10,12-pentaacetyl-2,4,6,8,10,12-hexaazaisowurtzite (4-(ethoxyacetyl)-2,6,8,10,12-pentaacetyl-2,4,6,8,10,12-hexaazatetracyclo [5.5.0.0^5,9^.0^3,11^]dodecane, **13c**). To evaluate their analgesic activity, the hot-plate test, abdominal constriction test, tail immersion test, and Randall–Selitto test were conducted, with Tramadol used as the reference drug due to its potent analgesic properties and opioid mechanism of action. During the acute toxicity evaluation of these compounds, no animal deaths were observed as monitored by veterinary professionals in outbred CD1 mice. 

In the abdominal constriction test, intragastric administration of compound **11c** resulted in a notable analgesic effect characterized by a pronounced “dome-like” pattern. Analgesic activity was observed at doses of 25 (44.7%), 50 (86.6%), 100 (48.4%), and 200 mg/kg (37.3%), effectively suppressing the development of writhing pain reactions in mice. Notably, 25% (50 mg/kg) and 12.5% (200 mg/kg) of mice treated with compound **11c** showed no pain reactions, which was not observed with Tramadol. Furthermore, at a dose of 50 mg/kg, compound **11c** exhibited a superior efficacy compared to the reference drug, as evidenced by the pain inhibition value (86.6% vs. 39.1% for Tramadol).

In the Randall–Selitto test, intragastric administration of compound **11c** as single and course doses at 25–200 mg/kg resulted in a significant increase in pain threshold and latency to pain development in outbred CD1 mice 1 h and 2 h post-injection. This effect was particularly prominent at a dose of 100 mg/kg. The analgesic effect of compound **11c** at the 100 mg/kg dose surpassed the effect of Tramadol at all observation time points. Furthermore, a percentage of animals treated with compound **11c**, both as single and course doses, showed no signs of pain response (50–62.5% vs. 25–32.5%). 

In the abdominal constriction test, compound **13a** at doses of 100 and 200 mg/kg exhibited a prominent analgesic activity comparable to Tramadol. The compound demonstrated efficacy even at a 50 mg/kg dose, reducing the number of writhes by 30.3% and significantly shortening the latency of pain response onset compared to the negative control. When the dose was increased to 100 mg/kg, the antinociceptive response further improved to 58.8%, and at 200 mg/kg, the efficacy was 50.9%. Tramadol exhibited a pain-relieving effect of 57.5%. 

During the Randall–Selitto test, the maximum pain-relieving effect of compound **13a** was observed in animals receiving a single intragastric administration at a 25 mg/kg dose. This dose resulted in a 1.9-fold increase in the pain threshold and a twofold increase in latency compared to the negative control. Notably, 54.5% of mice treated with this dose showed no pain manifestations, compared to 9.1% in the negative control group. With course administration of compound **13a** at a 25 mg/kg dose, its analgesic action was further enhanced. The pain threshold increased by 2.9-fold, latency increased by 3.7-fold, and 54.5% of mice exhibited no pain manifestations. These values were not significantly different from those observed in the tramadol group. 

The animal groups treated with compound **13a** at single doses of 50 and 100 mg/kg exhibited a statistically significant increase in the number of mice without pain reactions, comparable to the effect observed in the tramadol group. The course administration of compound **13a** at doses of 50 and 100 mg/kg demonstrated a pronounced effect. Specifically, the pain threshold significantly increased by 2.3 and 2.6 times and the pain response latency time increased by 2.7 and 3.1 times. Furthermore, the statistically significant number of mice without pain reactions (50%) was observed only in the animal group that received the 100 mg/kg dose. The observed effects were found to be comparable to those of tramadol. It can be concluded that compound **13a** exhibited statistically significant activity at a 25 mg/kg dose throughout all the observation hours and at 50 and 100 mg/kg doses after three daily administrations. These findings indicate that compound **13a** has a similar activity level to tramadol.

During the hot-plate test, compound **13a** demonstrated a significant antinociceptive action. The pain reaction development latency increased notably, and the number of mice without pain manifestations showed a significant increase compared to the negative control group. The maximum effect observed with compound **13a** reached 81%, which was significantly higher than the corresponding value for tramadol, which was 40.5%. 

During the acetic acid writhing test, compound **13c** demonstrated a dose-dependent effect within the 25–200 mg/kg dose range. The effect showed a “dome-like” pattern, reaching its maximum value at a dose of 50 mg/kg (74.7%).

The animals who received a minimum dose of 25 mg/kg were observed to have a statistically significant decrease in writhing by 1.7 times compared to the controls, which ensured a 40.9% inhibition of the pain response. The administration of the drug at a 50 mg/kg dose resulted in a 1.8-fold increase in latency time and a 3.9-fold decrease in writhing compared to the control. It should be noted that the action of **13c** at this dose was significantly higher than that of Tramadol, decreasing the number of writhes by 1.9 times. The further dose escalation to 100 mg/kg led to a slight decrease in the expressiveness of the effect, whereby the inhibition of the pain response was 64%, the number of writhes declined by 2.8 times while the latency time of the pain response development increased 1.8 times compared to the group of untreated mice. The 200 mg/kg dose had a statistically significant analgesic effect (60%) as it decreased the number of writhes by 2.5 times and extended the pain response development time by 2.4 times. 

The antinociceptive activity evaluation of **13c** in the Randall–Selitto mechanical hyperalgesia test demonstrated that the pain threshold value in the animal control group was 263.5 ± 44.2 g after 1 h of the single injection of the solvent and 264.3 ± 59.8 after a 3-day administration in 1 h of the last injection. Tramadol exhibited a pronounced analgesic activity after 1 h of the single and 3-day administrations, which was due to a 1.8- and 1.7-fold increase in the pain sensitivity threshold and due to a 1.9- and 1.8-fold increase in the latency time of the pain response development, respectively. It is noteworthy that the number of mice without pain manifestations increased to 27% in both observation periods. The changes in the basic parameters of the analgesic effect seemed to be a tendency when **13c** was administered as single 25 and 50 mg/kg doses. Alongside this, the number of mice without a pain response was found to rise following the administration of the compound at 25 (27%) and 50 mg/kg (20%) doses. 

The course administration of compound **13c** enhanced its efficacy at doses of 25 and 50 mg/kg, resulting in a 1.9-fold increase in the threshold (25 mg/kg), a twofold (25 mg/kg) and a 1.9-fold (50 mg/kg) increase in the latency time, and an increase in animals without pain manifestations to 18% (25 mg/kg) and 30% (50 mg/kg). 

Compound **13c** efficiently enhanced the pain sensitivity of mice to compressing pain when administered at 100 and 200 mg/kg doses after 1 h of a single administration through means of increasing the pain sensitivity threshold by 2 and 1.8 times and the pain response time by 2.1 and 1.9 times. Furthermore, it increased the number of mice without pain response to 40% in both cases.

After a course administration of compound **13c** at a dose of 100 mg/kg, a similar pattern of effect was observed. This included a 2-fold increase in the pain sensitivity threshold, an increase in the latency period of pain development by 2.2 times, and an increase in the number of mice with a 15-s exposure reaching 50% compared to the control group. In the animal group that received compound **13c** at a dose of 200 mg/kg, there was a tendency towards increased main indicators of analgesic activity. Additionally, the number of mice showing a maximum analgesic action reached 50%.

The totality of the obtained data allows for the conclusion that the newly synthesized **13c** has a pronounced analgesic effect comparable and/or exceeding that of Tramadol. The detected activity is realizable due to its inhibitory effect on the peripheral and central mechanisms of pain syndrome development at supraspinal, spinal, and peripheral levels of the pain sensitivity organization. 

Thus, the evaluation results for **13c** at a single and a 3-day per os administration within the 25–200 mg/kg dose range in the mechanical paw compression test on outbred CD1 male mice suggest a pronounced antinociceptive activity of **13c** comparable to that of Tramadol at all observation periods. 

The analysis of the results obtained from the study on the analgesic activity of compounds **11c**, **13a**, and **13c** in comparison to the reference drug tramadol in different tests for somatogenic pain, including the abdominal constriction test, Randall–Selitto test, and hot-plate test, leads to the conclusion that these newly synthesized compounds exhibit a significant antinociceptive effect, even after a single administration, within the dose range of 25–200 mg/kg. The observed antinociceptive effect of these compounds is comparable to, or even superior to, that of the reference drug, tramadol.

No animal death was observed in any of the tests, suggestive of the drugs being safe. It should be highlighted separately that no side effects typical of opioid and non-steroid anti-inflammatory drugs were documented in the experiments, which is particularly relevant to the studied hexaazaisowurtzitanes exhibiting anti-inflammatory activity. 

## 3. Anti-Inflammatory Activity of Hexaazaisowurtzitane Derivatives

Potential analgesic agents may exert inhibitory effects on the development of inflammatory reactions of any kind regardless of the nature of the damaging factor, phase, or stage of the process. When evaluating the anti-inflammatory action of an analgesic agent of any origin, it is reasonable to investigate its impact using models of acute exudative and chronic proliferative inflammation. In the studies overviewed herein, the following widely used agents with anti-inflammatory activity were employed as reference drugs for the research: diclofenac sodium (active ingredient in medications such as Voltaren, Cataflam, etc.) [33,34], which is used for inflammatory joint disease, neuralgias, myosalgias, post-traumatic pain syndromes, postoperative pains, migrainous strokes, and renal and biliary colic; and meloxicam (active ingredient in medications such as Mobic, etc.), which is used for inflammatory and degenerative joint diseases accompanied with pain syndrome and neuralgias [40,41].

A moderate anti-inflammatory effect of compound **10a** was revealed on models of carrageenan- and formalin-induced acute exudative inflammation and chronic immune inflammation [43].

Compound **11c** had a higher anti-inflammatory action compared to diclofenac sodium, as evidenced by the study conducted on models of acute exudative edema, carrageenin inflammation, and chronic proliferative inflammation in mice [51]. 

In the carrageenan-induced mouse paw edema model, the drug, when administered prophylactically into the stomach for three days, had a “dome-like” dose-dependent anti-exudative effect comparable to that of diclofenac both 1 h (25 mg/kg) and 3 h (25 mg/kg, 50 mg/kg) post-injection of phlogogen.

In the proliferative chronic inflammation model, the best result was observed at a dose of 12.5 mg/kg: a statistically significant anti-exudative activity (44.5%) was detected relative to the effect of diclofenac (8.7%), and the antiproliferative effect was 62.9%. The administration of **11c** at a dose of 25 mg/kg resulted in a statistically significant decrease in the crude granuloma burden by 1.9 times, dry granuloma burden by 2.2 times, granulomatous fibrotic tissue burden by 2.6 times and exudate burden by 1.7 times, whereby the exudation suppression was 39.6% and the statistically significant decrease in proliferation processes was 61.4% compared to the control. It should be noted that the anti-exudative effect was statistically significantly superior to the activity of diclofenac, with a comparable antiproliferative effect. A similar picture was observed when **11c** was administered at doses of 50 and 100 mg/kg. The anti-inflammatory effect was realizable through the exudation inhibition increasing to 36.2% (50 mg/kg) and 38.1% (100 mg/kg), and through the reduction of proliferation expression of up to 60.6% (50 mg/kg) and 55.3% (100 mg/kg).

Thus, it can be concluded that **11c** has a pronounced dose-dependent effect with a considerable decline in the granulomatous infiltration and exudation under chronic proliferative inflammation. This compound at doses of 12.5–100 mg/kg was significantly superior in the anti-exudative effect to the reference drug diclofenac. Moreover, the two inflammation models of different etiologies proved **11c** to have an anti-exudative action in mice and rats (male and female) when administered both prophylactically and therapeutically. 

## 4. Anticonvulsant Activity of Hexaazaisowurtzitane Derivatives

The presence of anticonvulsant action is of importance in neurologic and antishock therapies. In addition, anticonvulsant agents are used as first-line treatments for chronic or neuropathic pain. The reference drug employed in the conducted studies was carbamazepine, which is used for mania, prophylaxis of manic-depressive disorders, alcohol withdrawal syndrome, neuralgias of trifacial and glossopharyngeal nerves, diabetic neuropathy, and is an active ingredient in well-known antiepileptic medications such as Tegretol, Carbatrol, etc. [52,53].

The anticonvulsant effect of hexaazaisowurtzitanes was assessed on models of corazol-induced and thiosemicarbazide-induced seizures. Compound **10a** demonstrated an anticonvulsant effect that surpassed the efficacy of carbamazepine, showing the potential for application in neurological and neurosurgical practice [54].

The study on the anticonvulsant action of compound **11c** when administered intragastrically to outbred CD1 male mice at course doses of 25, 50, and 100 mg/kg was performed on a model of seizures induced by intraperitoneal injection of thiosemicarbazide (30 mg/kg). The results revealed a statistically significant increase in the latency time of clonic seizure onset when the compound was administered at a dose of 50 mg/kg. Additionally, compound **11c** exhibited efficacy when its anti-inflammatory action was tested on the model of carrageenan-induced edema in mice. Furthermore, compound **11c** at doses of 25 and 50 mg/kg showed an anticonvulsant effect on a model of seizures induced by subcutaneous injection of corazol at a 140 mg/kg dose. It extended the lifespan of mice and prolonged the latency time of the seizure onset. However, the observed effect was lower than that of the comparative drug, carbamazepine. 

## 5. Antihypoxic Activity of Hexaazaisowurtzitane Derivatives

In the treatment of acute circulatory disorders, drugs that improve the body’s utilization of oxygen and reduce its demand (enhancing the resistance to hypoxia) in the organs and tissues are widely used. One of the most well-known medications in this category is nootropil, which is often used as a reference drug in the biological screening for anti-hypoxic activity. It is prescribed for symptomatic treatment of intellectual and amnestic disorders, to reduce cortical myoclonia manifestations, in combination therapy of head injuries, ischemia and hypoxic manifestations to improve microcirculation [55,56].

The study [57] evaluated the antihypoxic activity of 4,10-dinikotinil-2,6,8,12-tetraacetyl-2,4,6,8,10,12-hexaazaisowurtzitane (4,10-dinikotinil-2,6,8,12-tetraacetyl-2,4,6,8,10,12-hexaazatetracyclo [5.5.0.0^5,9^.0^3,11^]dodecane, **11d**), 4,10-dinikotinil-2,6,8,12-tetraacetyl-2,4,6,8,10,12-hexaazaisowurtzitane as hydrochloride (4,10-dinikotinil-2,6,8,12-tetraacetyl-2,4,6,8,10,12-hexaazatetracyclo [5.5.0.0^5,9^.0^3,11^]dodecane as hydrochloride, 11d*), and 4,10-di(2-oxo-1-pyrrolidinecarbonyl)-2,6,8,12-tetraacetyl-2,4,6,8,10,12-hexaazaisowurtzitane (4,10-di(2-oxo-1-pyrrolidinecarbonyl)-2,6,8,12-tetraacetyl-2,4,6,8,10,12-hexaazatetracyclo [5.5.0.0^5,9^.0^3,11^]dodecane, **11e**) when administered prophylactically as a single dose on the acute tissue hypoxia model. The average lifespan of animals under acute tissue hypoxia was 762 ± 38 s in the control group and 888 ± 85 s in the comparison group. 

The per os administration of 11d* at doses of 2 mg/kg and 200 mg/kg considerably extended the lifespan of animals under acute tissue hypoxia to 830 ± 92 and 960 ± 110 s, respectively, which was 9% and 26% higher than the control group. The administration at a 200 mg/kg dose extended the lifespan of animals under acute tissue hypoxia by 8% compared to the comparison group.

The per os administration of **11d** at doses of 10 mg/kg and 100 mg/kg considerably extended the lifespan of animals under acute tissue hypoxia to 910 ± 88 and 900 ± 107 s, respectively, which was 19% and 18% higher than the control group.

The study found that the administration of compound **11d** at all tested doses (2, 10, 50, 100, and 200 mg/kg) resulted in an extended lifespan for the animals. This effect was comparable to the commonly used drug, nootropil. Interestingly, the improved solubility of compound **11d** in its salt form (specifically, as hydrochloride) did not significantly impact its biological action. 

The per os administration of **11e** at a dose of 2 mg/kg extended the lifespan of animals under acute tissue hypoxia to 797 ± 62 s, which was 5% higher than the control group. The dose escalation resulted in a decreased lifespan of animals under acute tissue hypoxia, demonstrating the prohypoxic action of the compound. 

The same study [57] evaluated the antihypoxic action of 4,10-di(4-chlorocynnamoyl)-2,6,8,12-tetraacetyl-2,4,6,8,10,12-hexaazaisowurtzitane (4,10-di(4-chlorocynnamoyl)-2,6,8,12-tetraacetyl-2,4,6,8,10,12-hexaazatetracyclo [5.5.0.0^5,9^.0^3,11^]dodecane, **11f**) and 4,10-di(4-methoxycynnamoyl)-2,6,8,12-tetraacetyl-2,4,6,8,10,12-hexaazaisowurtzitane (4,10-di(4-methoxycynnamoyl)-2,6,8,12-tetraacetyl-2,4,6,8,10,12-hexaazatetracyclo [5.5.0.0^5,9^.0^3,11^]dodecane, **11g**) on the model of normobaric hypoxia with hypercapnia (jar hypoxia). 

The average lifespan in the control group under jar hypoxia was 716 ± 16 s, and the average time between the first agonal breath and the animal deaths was 22 ± 4 s.

The per os administration of **11f** at doses of 10 mg/kg and 50 mg/kg considerably extended the lifespan of animals under jar hypoxia to 793 ± 6 and 896 ± 68 s, respectively, which was 10% and 20% higher than for the control group. The administration at a 10 mg/kg dose did not increase the time from the onset of agony to the whole death compared to the control group, whereas the 50 mg/kg dose administration led to a 63% increase in this parameter.

The per os administration of **11g** at a 10 mg/kg dose negligibly extended the lifespan of animals under jar hypoxia, whereas the administration of the test compound at a 50 mg/kg dose increased this parameter by 19% compared to the control group. The time between agony and death of the animals who received **11g** did not differ from the control. 

Thus, the antihypoxic activity of some hexaazaisowurtzitane derivatives was found to be on a par with commonly used drugs. 

## 6. Safety in Use of Hexaazaisowurtzitane Derivatives

Analgesic and anti-inflammatory agents are commonly used in daily medicinal practice; however, it is worth noting that not all of them are approved for use in oncologic patients. In oncology, pain manifests as a chronic pain syndrome, which is associated with the development of depressive disorders, suicidal intentions and actions, fear, and aggressive reactions, and undoubtedly requires treatment [58]. The confirmation of the safety of drugs for use in these patients is a mandatory and urgent objective [59,60,61]. Such studies have been conducted to evaluate the effect of compound **10a** on the growth and metastasis of subinoculated Lewis lung edema in C57Bl/6 mice. In those studies, cyclophosphamide was used as a reference drug. Cyclophosphamide is the active ingredient in medications marketed under trade names Cyclophosphane, Carloxan, and so on, which are used in the therapy of small cell lung cancer; ovarian cancer; cervical and uterine cancer; breast cancer; bladder cancer; prostate cancer; testicular seminoma; neuroblastomas; retinoblastomas; angiosarcomas; reticulosarcomas; lymphosarcomas; chronic lymphocytic leukemia and chronic myeloid leukemia; acute lymphoblastic, myeloblastic and monocytic leukemia; lymphogranulomatosis; non-Hodgkin’s lymphoma; multiple myeloma; Wilms tumor; Ewing’s sarcoma; soft tissue sarcomas; osteosarcoma; germ cell tumors; mycetoma; autoimmune diseases, including systemic conjunctive tissue diseases such as rheumatoid arthritis, psoriatic arthritis, autoimmune hemolytic anemia, nephrotic syndrome; and for suppression of transplant rejection responses [62,63].

It was discovered in a study conducted on mice with subinoculated Lewis lung carcinoma that the use of compound **10a,** when administered over a course of 2 to 21 days after tumor development, resulted in a 3.2-fold reduction in the area of metastatic lesions compared to the control group. The tumor burden in the group treated with compound **10a** exhibited a statistically significant decline compared to the control, with the tumor growth inhibited by 24%. Furthermore, when compound **10a** was prescribed in combination with cyclophosphan, a potentiation of the antimetastatic action was observed. The group of animals treated with the combination of cyclophosphan and compound **10a** showed a significant 12.8-fold decrease in the number of metastases compared to the group treated with cyclophosphan alone. Overall, the study demonstrated the safety in use of compound **10a** in oncology [64].

Based on the identified inhibitory effect on tumor growth, the potentiation of cyclophosphan in inhibiting the spread of the experimental tumor in animals, and the absence of gastrotoxicity when administered for chronic conditions, compound **10a** can be recommended for clinical evaluation as a choice agent for analgesia in oncologic patients. 

## 7. Conclusions

The design of new highly efficacious analgesics is a pressing issue in modern pharmacology because the analgesic drugs currently used in clinical practice do not meet all the efficacy and safety requirements (gastro-, nephron-, hepato- and haematotoxicity, negative impact on fertility, cardiovascular effects, genotoxicity and gonadotropic effects, addiction development, etc.). The scale of problems associated with the undesirable side effects of nonsteroidal anti-inflammatory drugs (NSAIDs) and the abuse of opioid analgesics as drugs for medicinal purpose has led to what is commonly known as the opioid crisis. A modern approach to treating pain conditions of various etiologies is to use highly selective agents capable of specifically blocking receptors that directly perceive pain stimuli and/or inflammatory mediators. Such substances include blockers of sodium ion channels, agonists of potassium ion channels, COX-3 inhibitors, blockers of proton sensitive ASIC3 channels, k-opioid receptors, antagonists and modulators of TRP ion channel receptors, NMDA receptor antagonists, mGluR5 receptor antagonists, and others. Non-narcotic analgesic drugs for the management of severe and moderate pain, which act on the central and peripheral nervous system as modulators of nerve impulse transmission and generation, and do not cause addiction or dependence, represent a relatively new direction in pharmacology as an alternative to opioids. Another modern direction in the development of potent non-narcotic analgesics, which act simultaneously on multiple biomolecular targets associated with the analgesic effect, is the creation of multitarget-directed drugs (MTDD).

In light of this, the development of new efficacious, low-toxicity, fast-acting analgesic agents that do not possess the side effects of nonsteroidal anti-inflammatory drugs and narcotic analgesics, for oral administration, such as encapsulated pharmaceutical dosage forms, is within the scope of the modern direction in the search for analgesic agents for the therapy of pain syndromes of various etiologies.

The discovery of the multitarget mechanism of analgesic action, as well as the anticonvulsant, anti-inflammatory, antitumor, and antihypoxic properties of the hexaazaisowurtzitane derivatives, along with their acceptable safety profile and pharmacokinetic parameters, are essential factors that warrant further research in this field and lay the groundwork for the development of a new class of medicinal drugs. 

Furthermore, the design of novel compounds within this class of hexaazaisowurtzitanes will considerably contribute to the progress in the chemistry of polycyclic cage amines that have a great potential for application in various fields. 

## Data Availability

Data are contained within the article.
